# Autologous Fat Grafting for Third-Degree Hand Burns: A Case Report With Patient and Observer Scar Assessment (POSAS)-Based Evaluation

**DOI:** 10.7759/cureus.101457

**Published:** 2026-01-13

**Authors:** Karen Rodríguez Franco, Juan Darío Alviar Rueda, Mónica Alexandra Ramírez Blanco, Camilo Andrés Granados González

**Affiliations:** 1 Plastic and Reconstructive Surgery, Hospital Universitario de Santander, Bucaramanga, COL; 2 Plastic Surgery, Universidad Industrial de Santander, Bucaramanga, COL; 3 Hand Surgery, Universidad Industrial de Santander, Bucaramanga, COL; 4 Plastic Surgery, Hospital Universitario de Santander, Bucaramanga, COL

**Keywords:** autologous fat graft, hand burn, hand reconstruction, microfat graft, patient and observer scar assessment scale (posas)

## Abstract

Wound management remains a fundamental component of plastic and reconstructive surgery [1]. Among the available treatment options, autologous fat grafting has gained attention due to its ability to support soft-tissue regeneration, enhance revascularization, and modulate immune responses through the secretion of bioactive molecules, cytokines, and growth factors [2]. It also provides anti-inflammatory, proangiogenic, and regenerative effects, which are particularly valuable in treating conditions affecting the hand and upper extremity [1,3]. Human adipose tissue represents a highly suitable implantable biomaterial owing to its rich reservoir of bioactive substances, such as extracellular matrix constituents, diverse growth factors, and stem or progenitor cell populations [2]. The present case illustrates how the use of autologous fat grafts as a biological dressing in a patient with a third-degree thermal burn and extensor tendon exposure in the hand resulted in favorable wound bed evolution and effective preparation for subsequent skin grafting. We present the case of a 47-year-old female patient with a 25 cm² soft-tissue defect on the dorsum of the left hand secondary to a third-degree thermal burn caused by contact with a metal sheet, with tendon exposure of the extensor mechanism of two fingers. The defect was managed using decanted autologous fat grafts, without centrifugation, serving as a “bioactive scaffold.” Tissue quality was evaluated pre- and postoperatively using the POSAS (Patient and Observer Scar Assessment) scale and photographic comparison. The treatment resulted in defect contraction and healthy granulation tissue formation, with satisfactory clinical progression documented through images and scar assessment scores. Positive changes were observed in the POSAS scale, with improvements in vascularity, pigmentation, elevation, surface roughness, tissue flexibility, and wound surface area, including significant contraction of the wound and reductions in pain and pruritus. No complications were reported. Vascularity improved by 70% compared to the initial assessment, while pigmentation, volume, and roughness each improved by 50%. Flexibility showed a 60% improvement.

The functional capacity of the hand was evaluated at the fourth postoperative month, showing a QuickDASH score of 2.3/100, indicating adequate hand rehabilitation [4]. Autologous fat grafting represents a safe, accessible, abundantly sourced, low-morbidity, simple technique to obtain, and a cost-effective surgical option for the treatment of complex wounds, particularly in the hand [5]. Its regenerative, angiogenic, and anti-inflammatory properties promote high-quality tissue formation, enhanced granulation, and wound contraction, ultimately improving both functional and aesthetic outcomes. In the clinical case presented, fat grafting enabled effective tendon coverage and preparation of the wound bed for skin grafting, as confirmed by objective improvements in POSAS scores. These findings highlight its potential for broader clinical application and integration into wound care protocols.

## Introduction

Wound management remains a fundamental component of plastic and reconstructive surgery [1]. There is a growing clinical and scientific interest in applying tools that accelerate healing processes and reduce complications and the costs of wound management [2]. In this sense, the emergence of the use of autologous fat grafts has been positioned in the last decade as an innovative alternative that expands therapeutic options [3]. Its widespread use is based on its ability to support soft tissue regeneration, enhance revascularization, and modulate immune responses through the secretion of bioactive molecules, cytokines, and growth factors that are particularly valuable in treating conditions of the hand and upper extremity [1-4]. Taking this into account, the aim has been to describe its effect on patients in the Colombian context in order to have evidence on accessible techniques. This case report describes the management of a patient with a coverage defect resulting from a third-degree thermal burn with tendon exposure, successfully treated with autologous microfat grafting. The quality of the tissues before and after the procedure is assessed using the POSAS (Patient and Observer Scar Assessment) scale, evaluating vascularity, pigmentation, volume, and surface roughness, along with photographic comparison, all of which demonstrated satisfactory outcomes. Additionally, functional assessment was performed using the quickDASH scale, which demonstrated adequate rehabilitation without sensory disturbances or limited range of motion of the fingers.

## Case presentation

A 47-year-old woman with no relevant medical or traumatic history presented to the emergency department of a tertiary-care hospital in Santander, Colombia, after sustaining a third-degree burn on the left hand. The injury occurred following direct contact with a heated metal sheet, resulting in a thermal burn.

**Figure 1:**
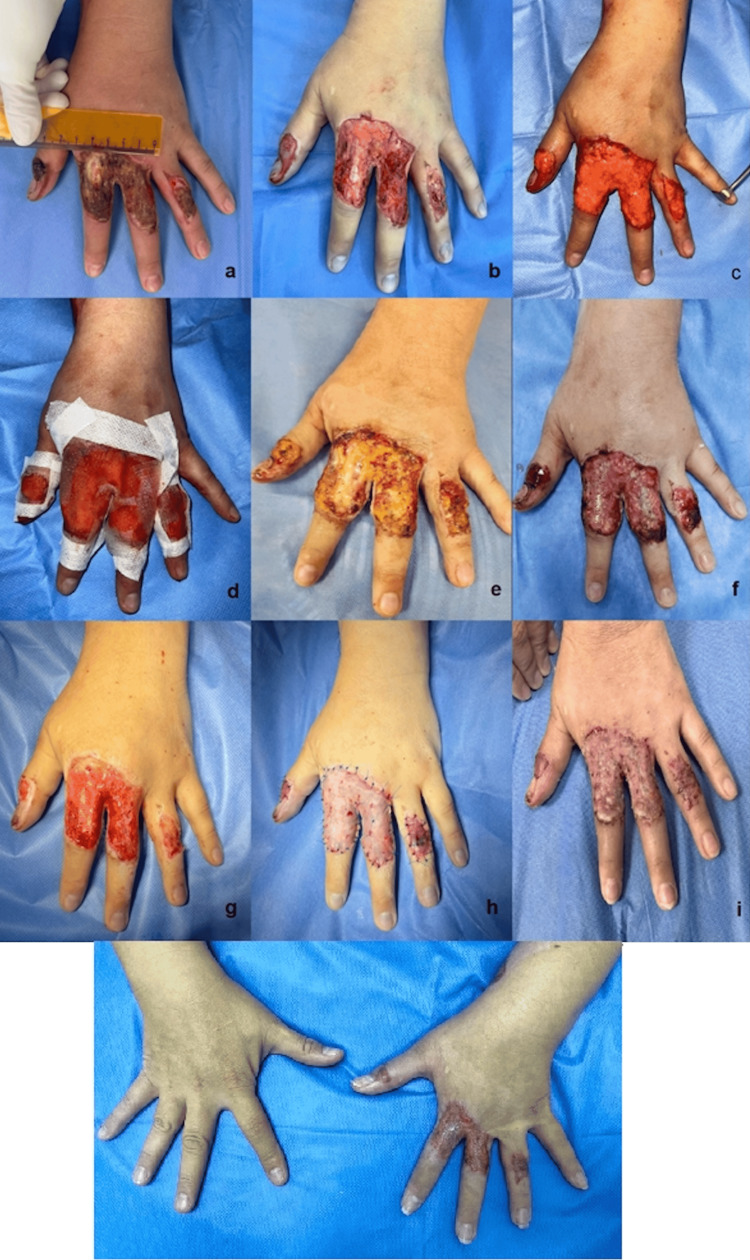
Clinical evolution of a third-degree burn treated with autologous microfat grafting. a. Third-degree thermal burn measuring 25 cm² on the extensor surface (zones TII, II, III, IV) of digits 1, 2, 3, and 4 of the left hand. b. Escharotomy performed on day eight post-injury. c. Application of non-centrifuged autologous fat grafts on day 10 posttrauma. d. Coverage with a non-adherent paraffin dressing. e. First dressing change on postoperative day three. f. Postoperative day seven: healthy granulation tissue observed, with complete coverage of the extensor tendon of the fourth digit and partial coverage of the extensor tendon of the second digit. g–h. Postoperative day eight: coverage with intermediate-thickness skin grafts. i. Partial skin grafts in the process of integration, week three. j. Postoperative week eight, skin grafts fully integrated without pathological scarring, full range of motion of the fingers

On physical examination, the left upper limb demonstrated preserved superficial and deep flexion, as well as intact extension, abduction, adduction, and digital opposition. Sensory function remained intact distally, and capillary refill was 2 seconds. However, inspection of the dorsum of the left hand revealed a 25 cm² soft-tissue defect with exposed extensor tendons of the first, second, third, and fourth digits, corresponding to extensor zones TII, II, III, and IV (Figure 1, letter b). No additional abnormalities were identified.

Interventions

The initial treatment consisted of surgical debridement of the wound with escharotomy on the eighth day, followed by clinical reassessment and planning for autologous fat grafting to provide coverage of the extensor tendon with healthy granulation tissue. On the 10th day, autologous fat micrografts processed by sterile decantation (without centrifugation) were applied to the wound bed. Approximately 2 cc of graft per cm² was placed over the wound as a dressing, functioning as a biological scaffold. A non-adherent paraffin-impregnated dressing was used to cover the treated area. Photographic documentation and POSAS (Patient and Observer Scar Assessment Scale) scores were obtained before and after the procedure.

During follow-up, by postoperative day seven, healthy granulation tissue was observed, with complete coverage of the extensor tendons of the second and third digits (Table 1). Pre- and postoperative assessment using the POSAS scale demonstrated improvement across all evaluated domains-vascularity, pigmentation, volume, surface roughness, and flexibility. Photographic comparison also confirmed the 24% contraction of the initial defect, with a wound bed prepared to be grafted with partial skin grafts, whose integration proceeded without complications.

**Table 1:** POSAS scale application: preoperative and postoperative assessment (observer format) The results showed that by postoperative day 14 in this case, vascularization showed a 70% improvement compared to the initial evaluation according to the POSAS scale. Improvements in surface roughness, pigmentation, and volume of the wound bed were recorded at 50%, while enhancements in flexibility reached 60% (Table 1). The autologous fat graft effectively functioned as a bioactive scaffold, optimizing wound bed preparation before the partial skin graft, further reducing the required size of the graft due to a 24% wound contraction. Photographic evidence revealed progressive and adequate graft integration.

Aspect	Preoperative	Postoperative	Change (Delta)
Vascularity	10	3	7 (70%)
Pigmentation	9	4	5 (50%)
Volume	8	4	4 (50%)
Roughness	9	4	5 (50%)
Flexibility	10	4	6 (60%)

## Discussion

Traditionally, soft tissue defects have been addressed using skin grafts or flap procedures, without considering regenerative medicine. In recent years, autologous fat grafting has gained prominence as a valuable alternative due to its minimally invasive nature, its ability to restore tissue volume, and its inherent regenerative capacity. These characteristics make it particularly advantageous for managing hand coverage defects [5]. Studies suggest that adipose tissue can not only function as a volumetric filler but can also modulate the biochemical properties of the skin through its immunomodulatory, angiogenic, anti-apoptotic, extracellular matrix remodeling, and multilineage cell capabilities [6,7]. However, there is currently no consensus regarding the automation of the fat grafting procedure.

Procedure

Donor Site

Different donor site options have been described, most commonly the abdomen, thigh, and knee, harvested using hand-held syringe aspiration [8]. The abdominal area is the most used donor site, demonstrating a higher density of stem cells derived from adipose tissue, according to previously conducted surveys [9]. In our case, we perform fat aspiration from the periumbilical region using a 3 mm grating cannula, which is consistent with the existing literature, to get microfat and maintain cell viability [8].

Harvesting

Multiple methods for fat harvesting have been described, and the literature continues to debate which technique provides the highest density and functionality of adipocyte-derived cells [10]. When reviewing the impact of manual syringe aspiration, suction-assisted lipectomy, and ultrasound-assisted lipectomy, no differences have been shown in cell viability and adipocyte functionality [7]. However, this increases the costs of the protocol, and this technology is not always available to carry out surgical procedures.

Manual syringe aspiration with lower mechanical vacuum has been associated with a higher adipocyte count and viability. Meanwhile, pre-liposuction infiltration with tumescent solution reduces trauma to the donor tissue [9]. In our case, under local anesthesia with a solution of 1000 cc of 0.9% normal saline and 1 ml of epinephrine in a 1:1 ratio relative to the volume to be extracted from the donor area, fat was extracted using 50 cc syringes, creating a 4 cc vacuum in each, obtaining 50 cc of fat micrograft. This required only one procedure and application for this patient. Other protocols have documented the requirement of an average of two applications to achieve the desired coverage [11]. 

Processing Techniques

The literature describes various processing methods, among which centrifugation, cotton gauze rolling, gravity separation, decantation and sedimentation, washing, and filtration are highlighted [9]. Several variations of the original method developed by Coleman have been described, and multiple studies report different centrifugation parameters [11]. However, some studies suggest that centrifugation should be avoided to maintain the full metabolic effect of the tissue and to reduce cell lysis [12]. For this reason, we eliminated this step in the graft processing and proceeded with mechanical decantation. The decantation process was performed by placing the lipoaspirate into sterile, empty 10-cc syringes for 20 minutes. The oil and aqueous layers are then discarded, and the fat layer is extracted for application.

On the other hand, cotton gauze rolling is an economical and low-trauma technique for processing fat grafts, in which the lipoaspirate is placed on gauze and gently rolled to absorb excess oil, blood, and tumescent solution. The process takes approximately 2 to 4 minutes and yields a more concentrated graft, increasing the vascular stromal fraction contained [11].

Lipoaspirate may also be prepared through washing and/or filtration, generally performed within a closed system [12]. This type of processing was not necessary because the size of the cannula's fenestrations ensures the expected particle size is obtained. Additionally, fat washing is primarily performed in macrofat handling to more effectively remove blood and injected solution, which is not applicable in this case.

When analyzing the type of application, whether subdermal, intramuscular, intradermal, or as a biological dressing, the choice will be made based on the expected effect. Intradermal and subdermal applications are preferred for remodeling healing processes with completed coverage, intramuscular applications to increase collateral vascularization, and biological dressings when there are still exposed structures requiring coverage. Cohort studies found that complete wound closure is achieved between 8 and 12 weeks postoperatively (POP), with no evidence of adverse events when applied as a biological dressing [13].

Applicability

Fat grafting is used in a wide range of clinical scenarios, most notably breast reconstruction, management of scars, wounds, burns, radiodermatitis, HIV-related lipodystrophy, and various aesthetic procedures [14]. In burn trauma, thermal injury frequently results in loss of subcutaneous tissue and disrupted wound healing, characterized by fibrosis and hypertrophic scarring, which can significantly limit hand mobility [7]. In this context, fat grafting has demonstrated multiple therapeutic benefits due to the qualities already described.

Additionally, it's worth mentioning that this option is a biocompatible tool, since using the patient's own tissue minimizes the risk of rejection and complications associated with exogenous materials [15]. Additionally, it is a readily available and cost-effective resource.

Furthermore, by accelerating tissue regeneration processes, it allows for a faster recovery compared to more invasive reconstructive procedures, reducing hospital stays and the emotional distress experienced by patients [16].

Clinical evidence supports its role in improving patient satisfaction, physical function, and chronic symptoms [17]. Documented effects include promotion of neovascularization, reduction of local inflammation, and attenuation of pain [9,15].

Assess the outcomes

The POSAS scale has undergone validity studies (reliability, internal consistency, content validity, concurrent validity, and clinical feasibility). It has shown adequate correlation with clinometric studies [18].

Tests with patients have demonstrated that a single observer is sufficient for a reliable assessment [18], providing an ordinal measure that correlates with parameterized and serial photographic records, which also allows for the objective measurement of changes in wound surface area, offering a comprehensive measure of both aesthetic and functional recovery following treatment.

## Conclusions

Autologous fat grafting emerges as a valuable and biologically active tool in the reconstructive management of third-degree burns of the hand, particularly when tendon exposure limits the use of conventional techniques and when early wound bed preparation is essential. In this case, the application of decanted, non-centrifuged adipose tissue provided a favorable microenvironment that promoted robust granulation, defect contraction, and complete tendon coverage within a short postoperative interval, demonstrating the capacity of adipose tissue to regulate inflammation, stimulate angiogenesis, and enhance the quality of soft-tissue regeneration.

Objective evaluation using the POSAS scale substantiated the clinical findings, revealing significant improvements across vascularity, pigmentation, tissue volume, flexibility, and surface texture. These outcomes align with the growing body of evidence supporting adipose tissue as a bioactive scaffold capable of modulating cellular responses and optimizing wound remodeling. The observed improvements translated not only into a wound bed suitable for successful split-thickness skin grafting but also into early functional recovery, as demonstrated by favorable QuickDASH scores at follow-up. From a practical standpoint, autologous fat grafting represents a safe, reproducible, minimally invasive, and cost-effective adjunct in the reconstruction of complex burn wounds of the hand. Its low morbidity, wide availability, and ease of intraoperative preparation position it as an attractive therapeutic option that can be integrated into both early and delayed reconstructive algorithms. Importantly, no complications were observed in this case, reinforcing its safety profile.
